# Research trends on lactate in cancer: a bibliometric analysis and comprehensive review (2015–2024)

**DOI:** 10.3389/fimmu.2025.1587867

**Published:** 2025-05-09

**Authors:** Sinong Li, Ziyi Yang, Mutian Lv, Lin Zong, Yihan Xie, Zoujuan Cai, Ying Zhang, Zhongqing Wang, Zhe Liu, Liang Sang

**Affiliations:** ^1^ Department of Ultrasound, The First Hospital of China Medical University, Shenyang, China; ^2^ Department of Nuclear Medicine, The First Hospital of China Medical University, Shenyang, China; ^3^ Department of Neurosurgery, The First Hospital of China Medical University, Shenyang, China; ^4^ Department of Information Center, The First Hospital of China Medical University, Shenyang, China; ^5^ Department of Pancreatic-Biliary Surgery, The First Hospital of China Medical University, Shenyang, China

**Keywords:** lactic acid, cancer, bibliometric analysis, VOSviewer, CiteSpace

## Abstract

**Objective:**

A bibliometric approach was employed to systematically analyze the trends and potential future developments in lactic acid-related cancer research over the past 10 years.

**Method:**

We conducted a bibliometric analysis of literature on lactic acid in cancer research from 2015 to 2024, using data collected from the Web of Science database. A bibliometric analysis was conducted to identify general research directions and trends in current publications, as well as to determine the most prolific and influential authors, institutions, countries, and keywords in lactate and cancer research. The data were collected and analyzed using VOSviewer (Leiden University, Leiden, Netherlands), Microsoft Excel (Microsoft, Redmond, Washington, USA), CiteSpace, and Biblioshiny, with a focus on analysis and visualization.

**Results:**

A total of 5,999 publications were analyzed, focusing on various aspects of the relevant literature, including year of publication, country, institution, author, journal, category, keywords, and research frontiers. The analysis of these publications reveals a general upward trend in publication volume from 2015 to 2024, with China and University of California System emerging as the most prolific country and institution, respectively. *SCIENTIFIC REPORTS* is the most frequently published journal, while *Oncotarget* is the most cited journal in the field. Zhang Y. was the most prolific author, publishing 100 documents over 10 years, with the highest citation count and an H-index of 28.Keyword analysis revealed five key themes in lactate-cancer research (2013–2023): Metabolic-epigenetic crosstalk, Tumor immunosuppressive microenvironment, Innovative therapies/drug delivery, Lactate-mediated signaling, Metabolic-targeted treatment strategies. Current research emphasizes the application of lactic acid metabolism in metabolic intervention, immune microenvironment regulation, combination of new therapeutic techniques and applications in specific cancer types.

**Conclusion:**

Research on lactic acid in cancer is growing rapidly, with China at the forefront of this field. Research into lactic acid’s role in immune cell regulation, metabolism, and signaling pathways, combined with multi-modal imaging, big data analytics, and innovative drug delivery, is set to become a key trend in future studies, which promises new directions for identifying therapeutic targets, biomarkers, and developing advanced treatments.

## 1 Introduction

Lactic acid (C_3_H_6_O_3_) is a product of glycolysis, typically catalyzed by the enzyme lactate dehydrogenase, which converts pyruvate into lactate. Under hypoxic conditions, intracellular lactate concentration increases and can be oxidized back to pyruvate, which enters the tricarboxylic acid (TCA) cycle to provide energy to the cell. Historically, lactic acid was regarded as a mere byproduct and waste product of anaerobic glycolysis (1). The Warburg effect demonstrates that tumor cells preferentially produce lactic acid via glycolysis, even under well-oxygenated conditions, and that cancer cell proliferation heavily relies on anabolic pathways supported by an adequate energy supply (2, 3). In recent years, metabolic reprogramming has been recognized as a hallmark of tumor cells (4). Metabolic reprogramming represents an adaptive shift in the balance between anabolic and catabolic processes, enabling tumor cells to meet their elevated demands for biosynthetic materials and energy during development (5).

Tumor progression depends not only on cell proliferation but also on the construction of the tumor microenvironment (TME), where metabolic reprogramming enables tumor cells to thrive and survive in the nutrient-deprived conditions caused by rapid proliferation (6). In the TME, tumor cells produce large amounts of lactic acid through glycolysis, promoting cell proliferation, inhibiting apoptosis, enhancing drug resistance, and inducing immunosuppression, thereby facilitating tumor metastasis (7, 8). The lactate shuttle theory posits that lactate functions as an energy carrier, transferring between different tissues and cell types within the same tissue. Similarly, lactate is transported through the basement membrane in precancerous tumors, serving both as an energy source and as an oncogenic signal. It promotes tumor-mesenchymal cell interactions and acidifies the tumor microenvironment (TME), facilitating tumor growth (9, 10). Tumor cells are resistant to acidic environments, while non-tumor cells are inhibited by such conditions (11). An acidified TME significantly alters the expression of immunosuppressive genes in tumor-infiltrating myeloid cells, promoting immune escape by tumor cells (12, 13). Recent studies have shown that lactate plays a pivotal role in tumor-mesenchymal interactions, serving not only as a metabolic fuel but also as a signaling molecule that promotes tumorigenesis (14, 15). These signaling molecules represent a potential strategy for treating tumor malignancy (16–18).

Bibliometrics emerged at the turn of the 20th century as a quantitative method for evaluating and analyzing the literature within a specific discipline (19, 20). An analysis of the retrieved data—including authors, keywords, journals, institutions, countries, and references—provides an overview of key developments and emerging trends in the field (21). Over the past 10 years, research on the relationship between lactic acid and cancer has evolved from a relatively low-profile area to a prominent research hotspot. However, no studies have systematically examined the trends and research hotspots of lactic acid in cancer research using a bibliometric approach. To address this knowledge gap, this study aimed to objectively characterize the current status and research directions of lactic acid in cancer research through bibliometric analyses. Specifically, it sought to identify the major contributors, institutions, countries, and research priorities; review current research hotspots; and predict future trends and perspectives in the field.

## 2 Methodology and material

### 2.1 Data collection

An extensive search and analysis were conducted using the Web of Science Core Collection (WoSCC) database. The Web of Science (WoS) is one of the world’s largest and most comprehensive academic information platforms, encompassing over 9,000 academic journals across diverse scientific disciplines (22). The WoS database is globally recognized as a comprehensive repository of authoritative and influential academic journals. Therefore, the WoS Core Collection databases, including the Social Science Citation Index (SSCI) and Science Citation Index Expanded (SCIE), were selected as the data source for this study. Subject searches conducted in the Web of Science Core Collection (WoSCC) (**Supplementary Material**). After reviewing the relevant literature and excluding articles that were not pertinent to this study, a total of 5,999 articles were included for analysis. The retrieved data were collected on February 20, 2025, to minimize bias caused by daily updates. The search results were exported as ‘complete records and references,’ including titles, authors, institutions, abstracts, journals, publication dates, and other information. These were saved as a plain text file (download_.txt) and imported into VOSviewer software to check for duplicates.

### 2.2 Affiliations clarification

In the Web of Science (WoS) database, The University of Texas MD Anderson Cancer Center (UT MDACC) is indexed as an independent institution distinct from The University of Texas. To maintain consistency with the original data structure and ensure reproducibility, we retained this classification. Notably, UT MDACC operates both as a degree-granting academic unit of The University of Texas System and as a standalone cancer research entity, which may explain its separate designation in bibliometric databases.

### 2.3 Data analysis

In this study, we utilized CiteSpace, VOSviewer, and RStudio’s bibliometric package for literature visualization and analysis, Microsoft 365 for table creation, Scimago Graphica for country mapping, and PowerPoint to design a flowchart of the literature screening process. CiteSpace, developed by Professor Chaomei Chen from Drexel University, is a free Java-based application designed for the visual analysis of trends and patterns in scientific literature (23). VOSviewer, developed by the CWTS Center at Leiden University in the Netherlands, offers robust functionality and a practical interface for co-occurrence and co-citation analyses (24). The number of publications, the average citations per publication, and the H-index were extracted from the Web of Science citation reports. The H-index, originally developed to measure a researcher’s scientific output, is based on the number of publications (N) that have been cited at least N times (25).

## 3 Results

**Figure 1:**
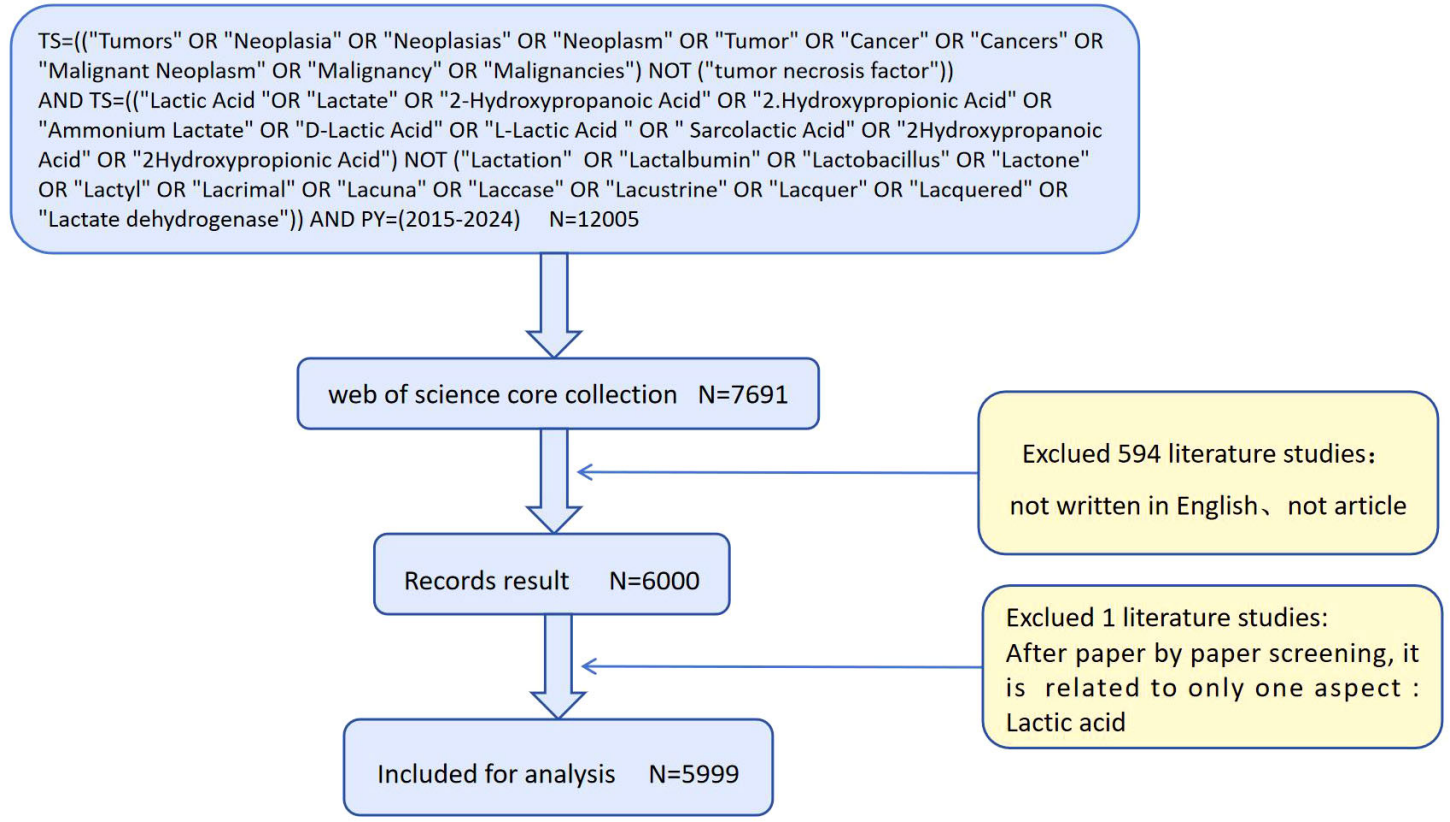
Process map.

The literature search resulted in a total of 5,999 records (**Figure 1**). The citation analysis conducted via the Web of Science (WOS) database identified a total of 144,775 citations. After excluding self-citations, the adjusted citation count stood at 134,266. These publications comprised 96,736 references, had an average citation frequency of 37.64, and collectively achieved 135 H-index values, appearing in 1,452 unique journals by 29,902 authors.

### 3.1 Publications

**Figure 2:**
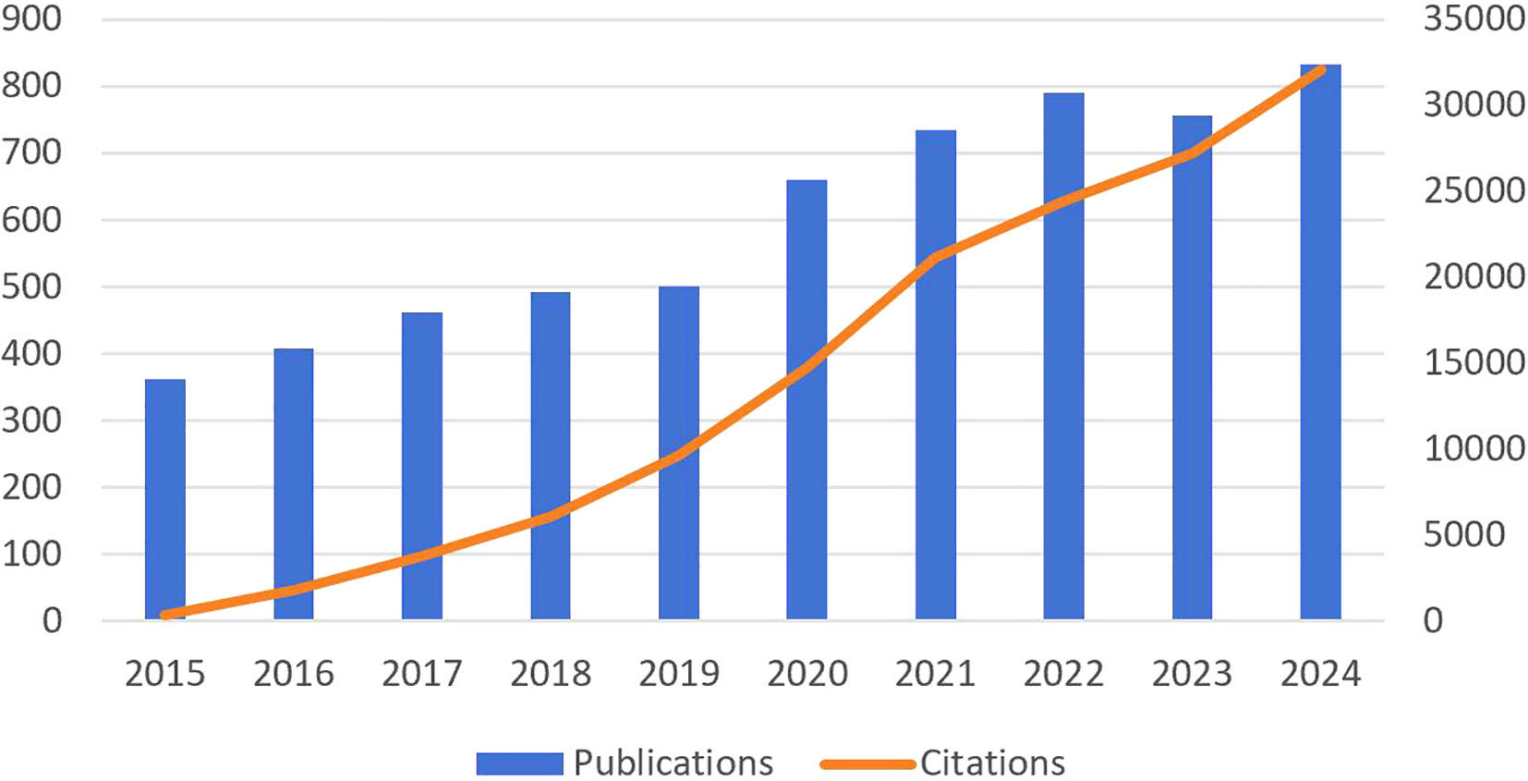
Trends in publications and citations.

This study included 5,999 relevant publications from WoSCC between 2015 and 2024. As of February 20, 2025, the included papers have garnered 144,775 citations, averaging 24.13 per paper, and collectively achieved 135 H-indices, according to the WOS citation report. The temporal distribution of publications exhibits a generally upward trend over the 10-year period, for citation volume 2014–2018 steady accumulation, the first citation inflection point occurs in 2019 and peaks in 2022. after 2023 the growth rate of issue volume slows down, but citation volume remains high (**Figure 2**).

### 3.2 Countries/regions

**Figure 3:**
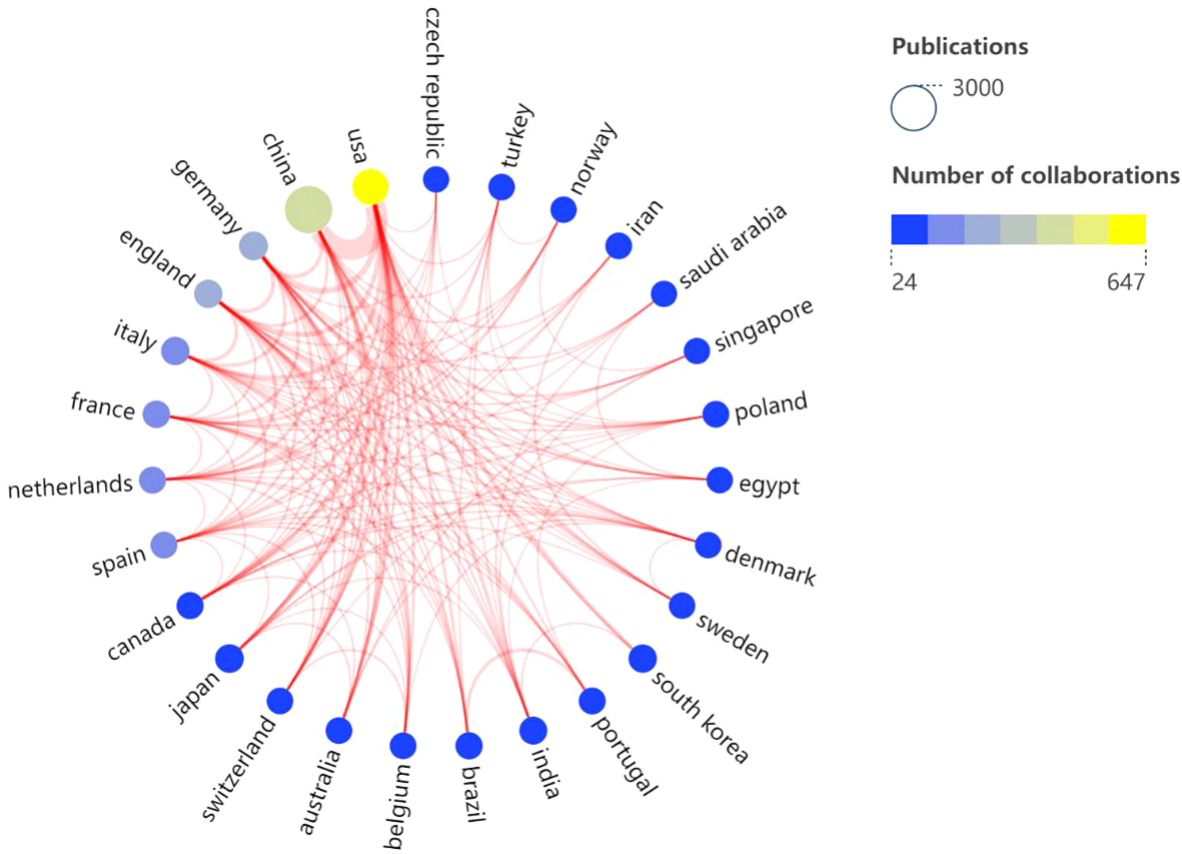
Visual analysis of the co-authorship of national publications. (Node size: the number of publications; Node color: the blue suggests the less the number of collaborations, the yellow suggests the more the number of collaborations, line thickness: the thicker the more cooperation).

Over the past 10 years, research articles focusing on lactic acid and cancer have been published across 108 countries/regions. China leads with 2,840 publications, followed by the United States (1,149), Germany (315), Japan (279), Italy (251), South Korea (244), England (224), India (195), France (158), and Brazil (128). In terms of citation counts, China is at the forefront with 62,840 citations, followed by the United States (46,181), Germany (8,691), England (7,493), and South Korea (7,091). Ranked by average citation count, the top countries are the United States (40.19), followed by England (33.45), France (29.92), South Korea (29.06), and Germany (27.59) (**Table 1**). Based on the SCImago Graphica Beta 1.0.26 analysis, the United States emerges as the most active participant in international collaborations, primarily partnering with China and Germany. Notably, the collaboration between China and the United States is the most extensive (**Figure 3**).

**Table 1:** Top 10 countries/regions in terms of number of publications, number of citations, and biased average number of citations.

Countries/Regions	Publications	Citations	Average of per publication
CHINA	2840	62,840	22.13
USA	1149	46,181	40.19
GERMANY	315	8,691	27.59
JAPAN	279	5,598	20
ITALY	251	6,271	24.98
SOUTH KOREA	244	7,091	29.06
ENGLAND	224	7,493	33.45
INDIA	195	3,641	18.67
FRANCE	158	4,728	29.92
BRAZIL	128	2,190	17.11

### 3.3 Institutions, journals, authors and press

#### 3.3.1 Institutions

A total of 5,214 institutions have published articles on lactate and cancer over the past 10 years. **Table 2** shows the top 10 institutions with the number of publications. Among these, University of California System leads in productivity with 178 publications, followed by the Shanghai Jiao Tong University (166) and the Chinese Academy of Sciences (165). The top five most cited institutions are the University of California System (9,211 citations), the University of Texas System (7,269 citations), the Chinese Academy of Sciences (6,110 citations), Shanghai Jiao Tong University (5,011 citations), and FuDan University (4,640 citations) (**Table 2**).

**Table 2:** Most relevant affiliations.

Affiliations	Articles	Citations	H index
UNIVERSITY OF CALIFORNIA SYSTEM	178	9,211	42
SHANGHAI JIAO TONG UNIVERSITY	166	5,011	41
CHINESE ACADEMY OF SCIENCES	165	6,110	36
FUDAN UNIVERSITY	153	4.640	36
UNIVERSITY OF TEXAS SYSTEM	135	7,269	40
SUN YAT SEN UNIVERSITY	125	3,765	32
NANJING MEDICAL UNIVERSITY	115	3,537	31
ZHENGZHOU UNIVERSITY	110	2,663	29
ZHEJIANG UNIVERSITY	106	3,127	30
CENTRAL SOUTH UNIVERSITY	88	1,875	26

#### 3.3.2 Journals

The 5,999 articles were disseminated across 1,452 journals. Among these, *Oncotarget* stands out with the highest H-index of 39, having published 99 related papers garnering 4,341 citations over the 10-year period. *SCIENTIFIC REPORTS, JOURNAL OF EXPERIMENTAL CLINICAL CANCER RESEARCH, PLOS ONE* and *CELL DEATH DISEASE* are also among the top five journals by impact. Notably, *SCIENTIFIC REPORTS* leads with the highest number of publications at 147, while *Oncotarget* has the highest citation count among the top ten journals by impact (**Table 3**).

**Table 3:** TOP10 source impact (by H index).

Journals	Publications	Citations	H index
ONCOTARGET	99	4,341	39
SCIENTIFIC REPORTS	147	4,329	38
JOURNAL OF EXPERIMENTAL CLINICAL CANCER RESEARCH	53	2,684	30
PLOS ONE	100	2,364	26
CELL DEATH DISEASE	45	1,925	26
BIOCHEMICAL AND BIOPHYSICAL RESEARCH COMMUNICATIONS	60	1,367	23
FRONTIERS IN ONCOLOGY	74	1,025	20
INTERNATIONAL JOURNAL OF MOLECULAR SCIENCES	108	1,090	18
CANCERS	83	1,020	18
NMR IN BIOMEDICINE	47	648	14

#### 3.3.3 Authors

A total of 29,902 authors contributed to the 5,999 publications. According to the Biblioshiny analysis of author impact (**Table 4**), Zhang Y. was the most prolific author, publishing 100 documents over 10 years, with the highest citation count and an H-index of 28.

**Table 4:** Author impact.

Authors	Articles	Citations	H index
Zhang Y	100	2,657	28
Wang Y	97	1,759	25
Li Y	96	2,177	26
Liu Y	79	1,465	22
Li J	72	2,101	25
Zhang J	67	1,924	23
Zhang L	60	1,342	22
Wang J	59	2,049	24
Wang L	56	1,365	22
Chen Y	55	1,523	19

### 3.4 Highly cited articles

**Table 5** lists the 10 most cited articles, with the highest citation count belonging to Zhang D ‘s study on *Metabolic regulation of gene expression by histone lactylation.* This study, published in 2019 in *Nature*, a highly prestigious and widely influential journal, for the first time, it was revealed that lactate directly catalyzes histone lysine lactylation (H3K18la), regulates gene expression and was demonstrated in macrophages that lactylation modifications drive M2-type polarization and promote cancer immunosuppression in a high-lactate microenvironment. The top 10 most cited articles investigate the crucial roles of lactic acid, in tumor development from diverse perspectives. These studies highlight the significant impact of lactic acid on tumor biology and elucidate the complexity of the associated metabolic networks.

**Table 5:** Top 10 cited articles (TC Total Citations).

Paper	Total Citations	TC per Year	Normalized TC
Zhang D, Tang Z, Huang H, Zhou G, Cui C, Weng Y, Liu W, Kim S, Lee S, Perez-Neut M, Ding J, Czyz D, Hu R, Ye Z, He M, Zheng YG, Shuman HA, Dai L, Ren B, Roeder RG, Becker L, Zhao Y. Metabolic regulation of gene expression by histone lactylation. Nature. 2019 Oct;574(7779):575-580.	1661	237.29	44.25
Hui S, Ghergurovich JM, Morscher RJ, Jang C, Teng X, Lu W, Esparza LA, Reya T, Le Zhan, Yanxiang Guo J, White E, Rabinowitz JD. Glucose feeds the TCA cycle via circulating lactate. Nature. 2017 Nov 2;551(7678):115-118.	1124	124.89	24.05
Faubert B, Li KY, Cai L, Hensley CT, Kim J, Zacharias LG, Yang C, Do QN, Doucette S, Burguete D, Li H, Huet G, Yuan Q, Wigal T, Butt Y, Ni M, Torrealba J, Oliver D, Lenkinski RE, Malloy CR, Wachsmann JW, Young JD, Kernstine K, DeBerardinis RJ. Lactate Metabolism in Human Lung Tumors. Cell. 2017 Oct 5;171(2):358-371.e9.	886	98.44	18.95
Angelin A, Gil-de-Gómez L, Dahiya S, Jiao J, Guo L, Levine MH, Wang Z, Quinn WJ 3rd, Kopinski PK, Wang L, Akimova T, Liu Y, Bhatti TR, Han R, Laskin BL, Baur JA, Blair IA, Wallace DC, Hancock WW, Beier UH. Foxp3 Reprograms T Cell Metabolism to Function in Low-Glucose, High-Lactate Environments. Cell Metab. 2017 Jun 6;25(6):1282-1293.e7.	800	88.89	17.12
Hensley CT, Faubert B, Yuan Q, Lev-Cohain N, Jin E, Kim J, Jiang L, Ko B, Skelton R, Loudat L, Wodzak M, Klimko C, McMillan E, Butt Y, Ni M, Oliver D, Torrealba J, Malloy CR, Kernstine K, Lenkinski RE, DeBerardinis RJ. Metabolic Heterogeneity in Human Lung Tumors. Cell. 2016 Feb 11;164(4):681-94.	792	79.20	18.85
Watson MJ, Vignali PDA, Mullett SJ, Overacre-Delgoffe AE, Peralta RM, Grebinoski S, Menk AV, Rittenhouse NL, DePeaux K, Whetstone RD, Vignali DAA, Hand TW, Poholek AC, Morrison BM, Rothstein JD, Wendell SG, Delgoffe GM. Metabolic support of tumour-infiltrating regulatory T cells by lactic acid. Nature. 2021 Mar;591(7851):645-651.	648	129.60	29.64
Davidson SM, Papagiannakopoulos T, Olenchock BA, Heyman JE, Keibler MA, Luengo A, Bauer MR, Jha AK, O’Brien JP, Pierce KA, Gui DY, Sullivan LB, Wasylenko TM, Subbaraj L, Chin CR, Stephanopolous G, Mott BT, Jacks T, Clish CB, Vander Heiden MG. Environment Impacts the Metabolic Dependencies of Ras-Driven Non-Small Cell Lung Cancer. Cell Metab. 2016 Mar 8;23(3):517-28.	587	58.70	13.97
Hu Y, Cheng H, Zhao X, Wu J, Muhammad F, Lin S, He J, Zhou L, Zhang C, Deng Y, Wang P, Zhou Z, Nie S, Wei H. Surface-Enhanced Raman Scattering Active Gold Nanoparticles with Enzyme-Mimicking Activities for Measuring Glucose and Lactate in Living Tissues. ACS Nano. 2017 Jun 27;11(6):5558-5566.	546	60.67	11.68
Chen F, Chen J, Yang L, Liu J, Zhang X, Zhang Y, Tu Q, Yin D, Lin D, Wong PP, Huang D, Xing Y, Zhao J, Li M, Liu Q, Su F, Su S, Song E. Extracellular vesicle-packaged HIF-1α-stabilizing lncRNA from tumour-associated macrophages regulates aerobic glycolysis of breast cancer cells. Nat Cell Biol. 2019 Apr;21(4):498-510.	530	75.71	14.12
Zhao H, Shang Q, Pan Z, Bai Y, Li Z, Zhang H, Zhang Q, Guo C, Zhang L, Wang Q. Exosomes From Adipose-Derived Stem Cells Attenuate Adipose Inflammation and Obesity Through Polarizing M2 Macrophages and Beiging in White Adipose Tissue. Diabetes. 2018 Feb;67(2):235-247.	474	59.25	12.29

### 3.5 Keywords

In this study, VOSviewer was employed to conduct a keyword co-occurrence analysis, thereby illustrating the distribution and characteristics of current research hotspots in the field of lactic acid and cancer. Themes were identified through keyword co-occurrence analysis using VOSviewer (v1.6.18), which automatically clustered high-frequency keywords (occurrence ≥68) into five distinct groups based on association strength. Each cluster’s thematic label was derived by manually evaluating its core keywords for conceptual coherence.

**Figure 4:**
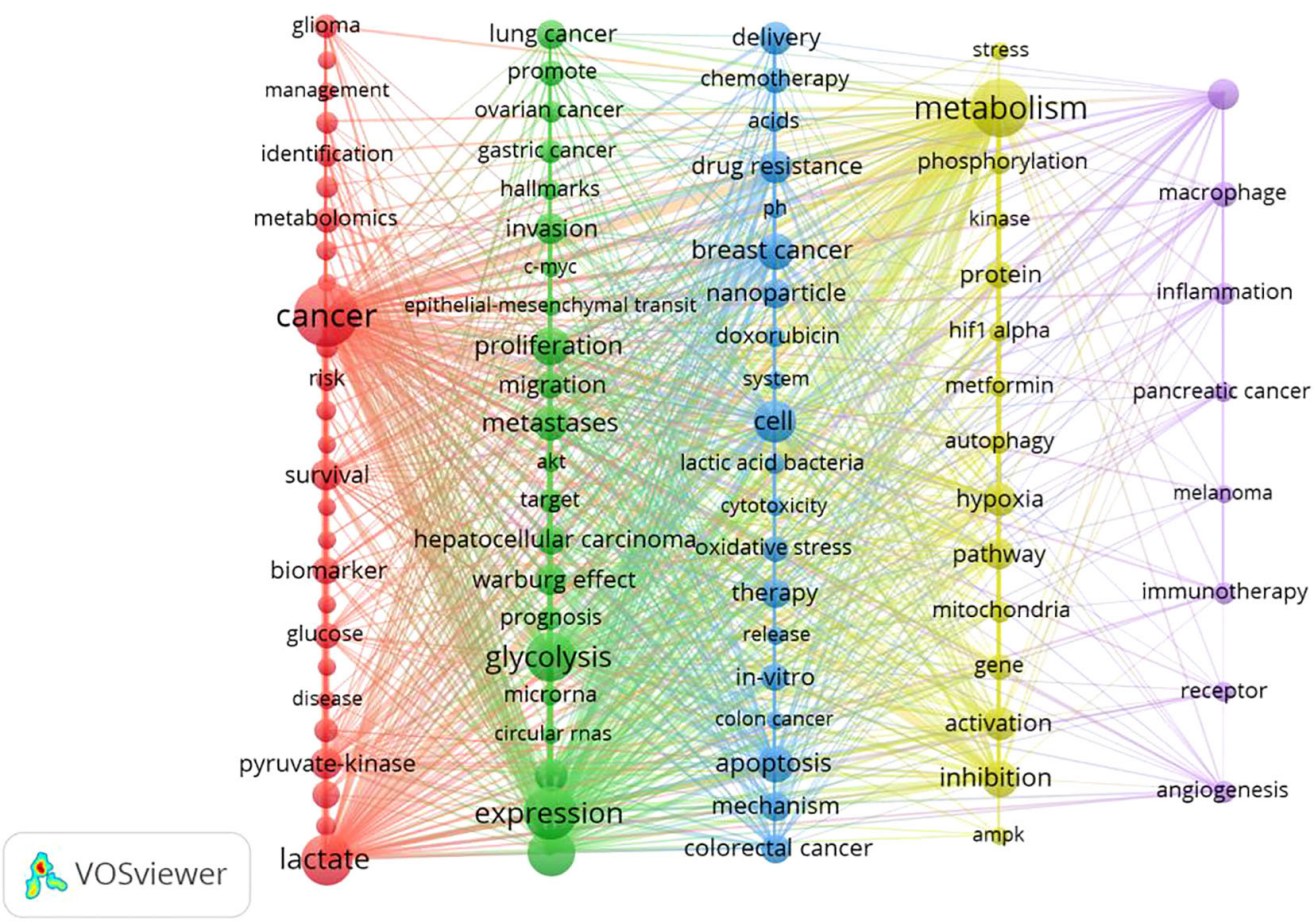
TOP25 keyword outbreaks (sorted by the start year of the outbreak). Co-occurrence analysis performed using Vosviewer.

**Figure 5:**
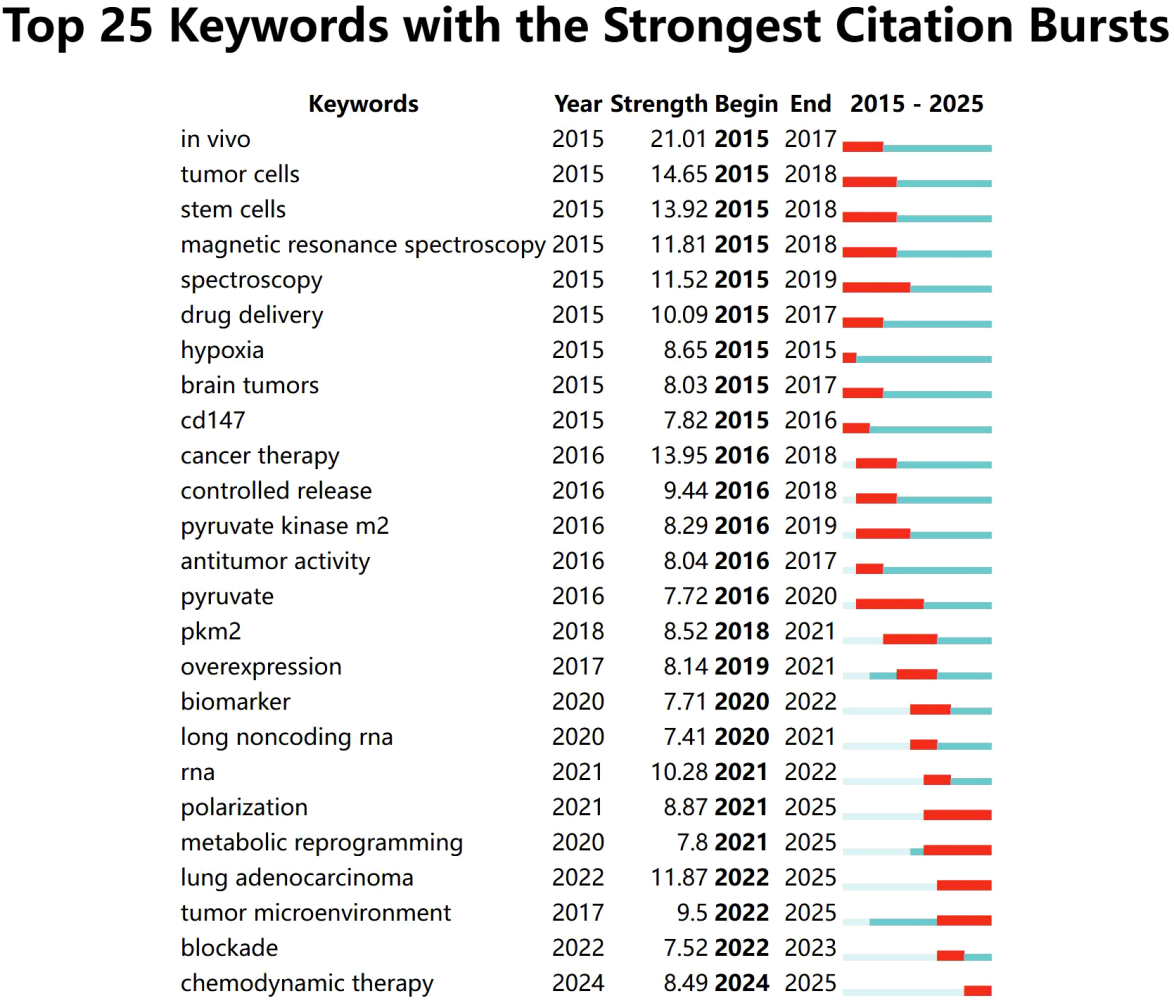
Keyword co-occurrence. (Node size: reflects keyword frequency; line thickness: Indicates co-occurrence strength; Cluster colors: Group semantically related terms).

**Figure 6:**
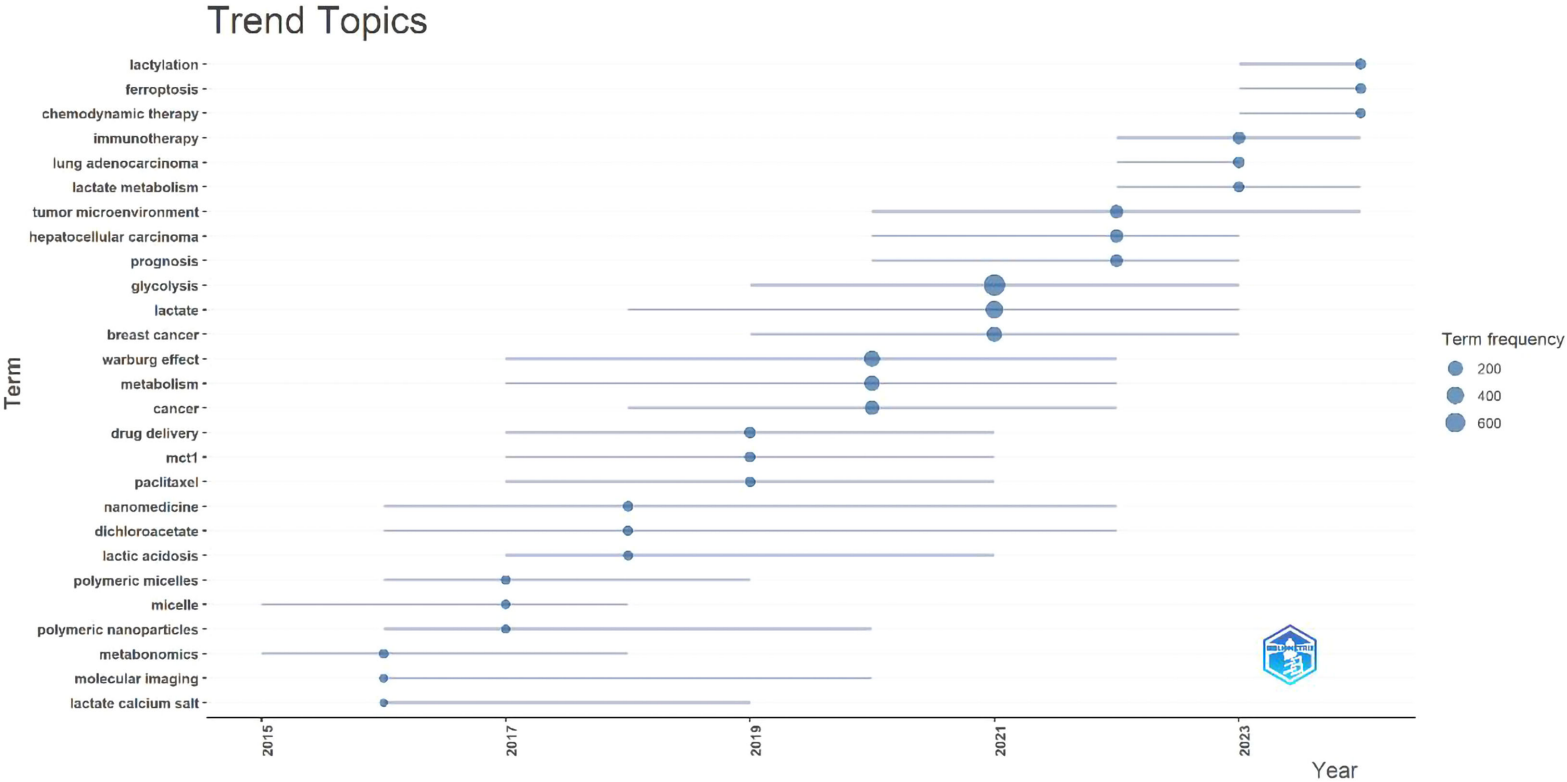
2015–2024 Topic trends. (Node size: reflects keyword frequency; Line length: The year in which the keyword appeared and ended).

A labeled visualization of keyword co-occurrences in lactate and cancer research was then generated (**Figure 4**) to identify research hotspots and core content. The keywords ‘cancer,’ ‘expression,’ and ‘metabolism’ emerge as the most prominent, suggesting that a substantial body of research is concentrated around these themes. The keywords included can be classified into the following six categories (**Table 6**, **Figure 5**): (1) Metabolic-epigenetic coaxial effects; (2) Tumor microenvironment-immunosuppressive network; (3) Innovative cancer therapy and drug delivery systems; (4) Lactate-mediated signaling cascades; (5) Metabolically Targeted Therapeutic Strategies, it can be concluded that the keywords ‘lung adenocarcinoma’, ‘tumor microenvironment’, ‘blockade’, and ‘chemodynamic therapy’ are key words that have appeared in the last three years (**Figure 4**). Based on the trend analysis of topics over the past 10 years (**Figure 6**), “lactylation”, “ferroptosis”, “immunotherapy”, “ lactate metabolism” and ‘breast cancer’ are terms that have grown significantly beyond 2019, suggesting that research on metabolic reprogramming in cancer is gaining interest, especially in lactate and related pathways; Terms such as “drug delivery” and “chemotherapy” also show steady growth, reflecting the continued efforts of researchers to refine and develop novel therapeutic strategies, it may also mean that more and more research is focused on personalized or targeted cancer therapies; “polymeric micelles” and “nanoparticles” have become increasingly popular in recent years, suggesting that researchers are More advanced drug delivery systems are being explored to improve drug efficacy or minimize side effects. Through the analysis of burst keywords, keyword clustering, and topic trends, it is possible to evaluate both past research focal points and predict future research directions in this field.

**Table 6:** Keywords cluster.

Items	Keywords
1	Biomarker;cancer; complex; dehydrogenase; diagnosis; differentiation; disease; glioblastoma; glioma; glucose; identification; *in-vivo*; lactate; magnetic resonance imaging; management; metabolite; metabolomics; model; mortality; mouse; nmr; prostate cancer; pyruvate-kinase; radiotherapy; risk; spectroscopy; survival
2	Aerobic glycolysis; akt; c-myc; circular rnas; epithelial-mesenchymal transition; expression; gastric cancer; glycolysis; hallmarks; hepatocellular carcinoma; invasion; lung cancer; metastases; microrna; migration; ovarian cancer; prognosis; proliferation; promote; target; tumor growth; warburg effect
3	Acids; apoptosis; breast cancer; cell; chemotherapy; colon cancer; colorectal cancer; cytotoxicity; delivery; doxorubicin; drug resistance; *in-vitro*; lactic acid bacteria; mechanism; nanoparticle; oxidative stress; ph; release; system; therapy
4	Activation; ampk; autophagy; gene; hif1 alpha; hypoxia; inhibition; kinase; metabolism; metformin; mitochondria; pathway; phosphorylation; protein; stress
5	Angiogenesis; immunotherapy; inflammation; macrophage; melanoma; pancreatic cancer; receptor; tumor microenvironment

**Figure 7:**
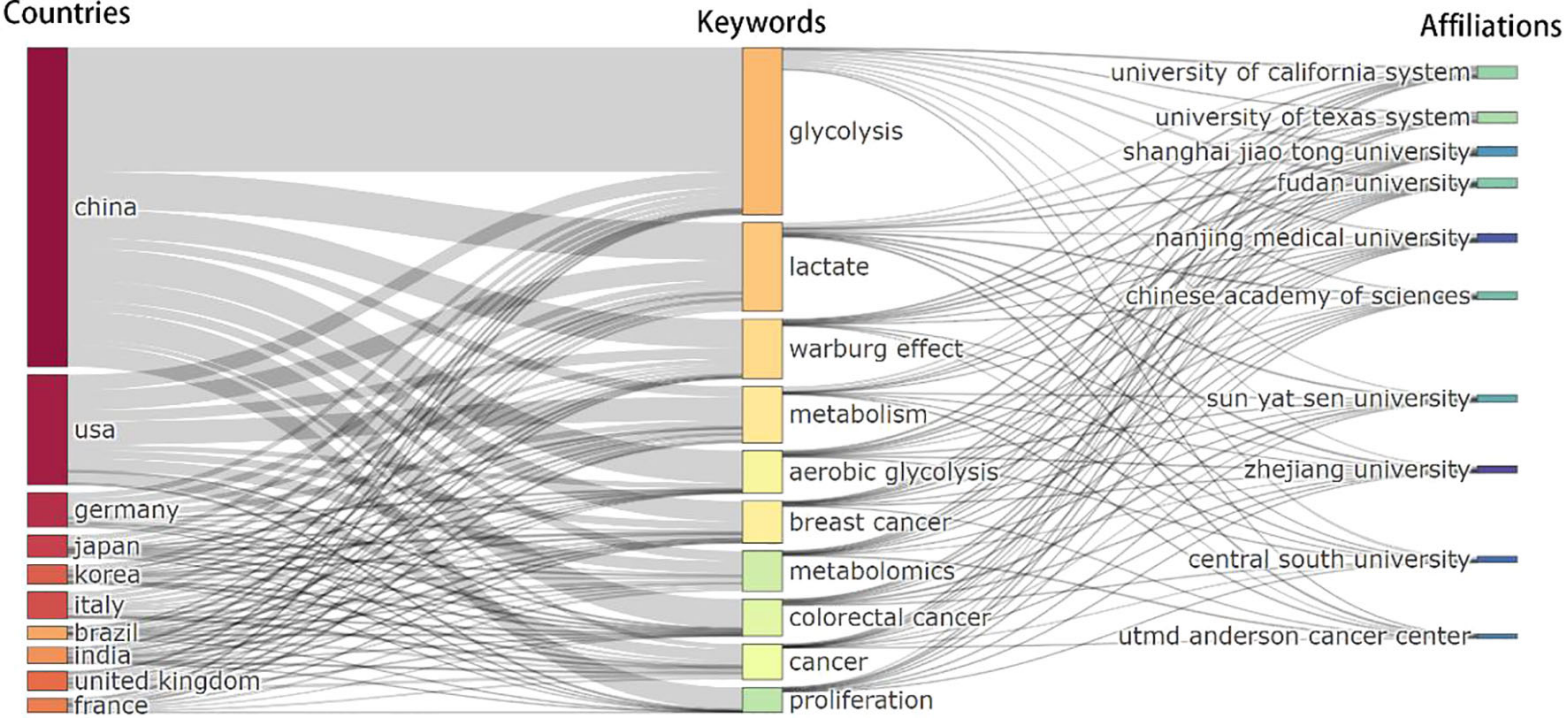
Global landscape of lactic acid and cancer research.

The joint analysis of countries, institutions, and keywords (**Figure 7**) clearly demonstrates the dominant role of China and the United States in lactate and cancer research, as evidenced by the prominence of core keywords and key institutions. The keywords ‘glycolysis’, ‘lactate’ and ‘Warburg effect’ serve as central hubs in this field of study, with relevance extending across multiple countries and institutions. Chinese institutions, including Shanghai Jiaotong University, Fudan University, Nanjing Medical University, Chinese Academy of Sciences, Sun Yat-sen University, Zhejiang University and Central South University have concentrated their research efforts on elucidating the underlying mechanisms associated with keywords such as ‘glycolysis,’ ‘Warburg effect,’ ‘lactate,’ and related terms. Institutions in the United States, such as the University of Texas System, the University of California System, and Utmd Anderson Cancer Center exhibit a well-balanced approach to research that integrates metabolic studies with clinical applications. Their investigative scope is notably broad, addressing a diverse array of topics that include ‘metabolism’ ‘cancer’ and ‘breast cancer.’ Researchers from other countries tend to focus on more specialized research pathways, highlighting terms like “aerobic glycolysis” and “metabolism” in their studies.

## 4 Discussion

This study represents the inaugural bibliometric analysis focusing on lactic acid within the context of cancer research, aiming to examine and evaluate the developmental trajectory and future prospects of this field over the past 10 years. The analysis reveals an upward trend in the number of publications over the past 10 years, with ZHANG Y. emerging as the most prolific author in this field. The most collaborative team comprised Kurhanewicz, John and Vigneron, Daniel B. Their primary research focus involved evaluating the role of lactate metabolism across various tumor types utilizing Hyperpolarized 13C MRSI technology. China emerges as the most productive country in this field, with UNIVERSITY OF CALIFORNIA SYSTEM leading institutional contributions. The journal publishing the highest number of articles on lactic acid in cancer research is *SCIENTIFIC REPORTS*. The most cited document in this field is titled *Metabolic regulation of gene expression by histone lactylation*. The visualization and analysis revealed a shift in research focus from traditional metabolic mechanisms to metabolic intervention, immune microenvironment regulation, combination of new therapeutic techniques and applications in specific cancer types. The role of lactate in the regulation of immune cells, its interplay with metabolic and signaling pathways, and the ongoing exploration of lactate metabolism, lactates, and related concepts are poised to emerge as a new focal point in future research. Moreover, the integration of tumor microenvironment-targeted drug delivery, immunotherapy, and chemodynamic therapy is anticipated to drive the future trajectory of cancer treatment. Consequently, the continued advancement of metabolic reprogramming, immunotherapy, and precision drug delivery systems is likely to constitute the core direction of future investigations.

### 4.1 Publications

From 2015 to 2024, the number of publications exhibited an overall upward trend and can be segmented into two distinct phases. The first phase is from 2015 to 2019, during which time the number of communications shows an increasing trend year by year. The significant growth, particularly from 2020 onward, indicates an increasing interest in this research area, likely driven by key advancements, including breakthroughs in lactate metabolism and tumor microenvironment mechanisms (26–32). In the second phase, from 2020 to 2024, the number of publications has stabilized at approximately 650, reflecting sustained research productivity in the field. Growth in the number of articles published in 2020 may be attributed to emerging hotspots in cancer metabolism research and significant technological advancements, including multi-omics approaches and metabolic targeting therapies (33, 34). Regarding the citation trend, citations grew rapidly from 2015 onward, peaking in 2024. This indicates that earlier research findings were extensively acknowledged and highly cited in subsequent studies.

### 4.2 Countries

As evidenced by the number of publications, citation counts, and average citations per country, China leads in publication volume. In contrast, the United States exhibits higher average citations per article, suggesting greater research influence, quality, and international recognition. Germany, England, and South Korea follow with the next highest numbers of publications and citations, indicating their mid-tier global standing and demonstrating steady growth in both research output and impact. The United States and China stand at the epicenter of international research collaboration, boasting the most extensive and densely interconnected cooperative networks, particularly between the two nations. Furthermore, the United States maintains close collaborative ties with notable European nations such as Germany and England. The United States has a wide range of collaborations across the globe, demonstrating that the strong combination of the United States and China is an important driver of development in the field, as well as the participation and contributions of other countries.

### 4.3 Affiliations

Among the top 10 academic institutions with the highest publication output in the field of lactate and cancer, eight are based in China, highlighting the country’s leadership in scientific productivity. The remaining two institutions are located in the United States. In addition, the institution with the most publications is the University of California System, which focuses on several key areas of research related to lactate, including: lactate-related epigenetic regulation (35), the relationship between the TCA cycle and lactate (36, 37) and metabolic adaptation of the immune microenvironment (38–40). Shanghai Jiaotong University, while being a secondary contributor in terms of publication volume, focuses on the relationship between lactate metabolism and tumor development, treatment and related mechanisms in lactate and cancer research, such as lactate metabolism and tumor mechanisms (41–44), Lactic acid and tumor therapy (45–48), Lactic acid and immunologic and chemotherapeutic sensitivity (49–52). Moreover, the organization has also conducted studies on lactic acid in different types of cancer, hepatocellular carcinoma (53, 54), pancreatic cancer (55, 56), breast cancer (57), lung cancer (58, 59).

### 4.4 Authors

Kurhanewicz, John, and Vigneron, Daniel B. formed a closely collaborating author cluster whose research focuses on evaluating the role of lactate metabolism in various tumor types using Hyperpolarized ^13^C MRSI technology. Their 2015 article in *NMR in Biomedicine*, titled *Real-time measurement of hyperpolarized lactate production and efflux as a biomarker of tumor aggressiveness in an MR-compatible 3D cell culture bioreactor* (60) has been cited 41 times. This journal is an international academic publication dedicated to the application of Nuclear Magnetic Resonance (NMR) technology in the biomedical field, with an impact factor of 2.7.

### 4.5 Documents

The most cited article, *Metabolic regulation of gene expression by histone lactylation*, highlights that lactate directly catalyzes histone lysine lactylation (H3K18la), regulates gene expression and was demonstrated in macrophages that lactylation modifications drive M2-type polarization and promote cancer immunosuppression in a high-lactate microenvironment. An analysis of the 10 most cited articles over the past 10 years highlights the pivotal role of lactate in tumor biology and its integration into complex metabolic networks, offering new insights into tumor metabolism.

### 4.6 Keywords

Keywords can help researchers identify emerging trends and grasp the direction of their research. The three most frequently occurring keywords were ‘cancer’, ‘expression’ and ‘metabolism’ indicating a primary focus on the role of lactic acid in cancer progression, epigenetic modifications, and tumor microenvironment regulation. An analysis of keyword relationships allows for their classification into four main categories: (1) Metabolic-epigenetic coaxial effects; (2) Tumor microenvironment-immunosuppressive network; (3) Innovative cancer therapy and drug delivery systems; (4) Lactate-mediated signaling cascades; (5) Metabolically Targeted Therapeutic Strategies. These clusters represent the primary focus areas of lactate and cancer research over the past 10 years.

Lactate, a central product of tumor metabolism, is intricately linked to the tumor microenvironment, including hypoxia, acidity, and immunosuppression, with metabolic reprogramming serving as a critical mechanism for microenvironmental regulation. For instance, lactic acid, a byproduct of cellular metabolism within the tumor microenvironment, facilitates cancer cell proliferation and invasion of the tumor microenvironment (61). In hypoxia-induced cancer cells, reduced LDHA expression leads to decreased LDHA activity, lactate production, and intracellular adenosine triphosphate (ATP) levels, significantly inhibiting colony formation and ultimately reducing cancer cell survival (62). Bok et al. also highlighted the dynamic nature of tumor metabolism by examining the role of lactate metabolism in prostate cancer metastasis (62). Moreover, tyrosine phosphorylation of PKM2 induces the Warburg effect (61), and phosphorylation of LDHA regulates redox metabolic homeostasis (63); both studies highlight the role of lactate in regulating the tumor microenvironment during metabolic reprogramming.

Lactic acid influences tumor progression during cancer invasion and metastasis by regulating extracellular matrix degradation and promoting cell migration, often resulting in increased treatment resistance and drug efficacy challenges. Studies have shown that lactic acid plays a key role in the signaling cascade mediating tumor progression. Research conducted by P. Jourdain et al. (64) revealed that L-lactate, generated through pyruvate metabolism, produces ATP and is released in an autocrine/paracrine manner. This process activates purinergic receptors and the PI3 kinase/P2Y/KATP signaling pathway, thereby counteracting glutamate-induced excitotoxicity and exerting neuroprotective effects. Additionally, lactate upregulates LDHB and MCT-1, converting lactate to pyruvate and generating ATP via the TCA cycle and oxidative phosphorylation (OxPhos), thereby driving tumor growth (65). In this study, the term pyruvate began to explode in 2016 and MCT-1 became a trending topic in 2019. However, lactic acid has also been shown to inhibit tumors. Zhang et al. (66) demonstrated that lactic acid creates a more acidic environment, favoring the selective conversion of α-ketoglutarate to L-2HG in tumors, which specifically induces tumor cell pyroptosis. The term metabolic reprogramming began to explode in 2021, lactic acid stimulates the secretion of IL-6 and VEGF by tumor-associated adipocytes through activation of GPR81, thereby further promoting tumor growth (67). Relevant studies suggest that targeting GPR81 and MCT-1 could offer a potential strategy for treating tumor malignancy (16–18).

Simultaneously, we analyze the frontiers, hotspots, and emerging trends in the research on lactic acid and cancer. Research on lactate and cancer focused on metabolic reprogramming and tumor microenvironment studies between 2015 and 2020. Since 2020, research has increasingly focused on functional mechanisms and clinical significance, with sustained growth in interest and relevance. In the past three years, keywords such as ‘metabolic reprogramming’, ‘polarization’, ‘lung adenocarcinoma’, ‘tumor microenvironment’, ‘blockade’ and ‘chemodynamic therapy’ reflect a shift in research focus from traditional metabolic mechanisms to metabolic intervention, immune microenvironment regulation (68–70), combination of new therapeutic techniques and applications in specific cancer types, such as lung adenocarcinoma (69, 71–73). In 2019, Zhang et al. (35) experimentally demonstrated that histone lysine lactylation (Kla), a novel *in vivo* post-translational modification, is derived from lactic acid. This modification represents a novel epigenetic mechanism linked to macrophage polarization, potentially affecting cancer progression and inflammatory disease pathways. Subsequently, terms such as polarization, metabolic reprogramming, and tumor microenvironment began to erupt in 2021. Although immune-related keywords have not appeared with significant frequency, the role of lactic acid in regulating immune cell function and the immune microenvironment is emerging as a prominent research focus. The synergistic effects of lactate with other metabolites and signaling pathways, such as Akt and HIF-1, in driving tumor progression are anticipated to become a prominent research trend. Furthermore, leveraging the metabolic properties of lactate to develop more targeted and efficacious therapeutic strategies may emerge as a promising trend in future research.

### 4.7 Limitations

In contrast to the traditional approach of reviewing numerous studies to summarize the current state of research on the link between lactic acid and cancer, bibliometric analysis provides a more intuitive and efficient method for acquiring information in this field. Researchers can selectively access the information they need from the article, thereby enhancing the efficiency of scientific research, staying updated on the latest field hotspots, and identifying emerging topics. However, this study has several limitations. First, the literature analyzed was limited to the SCI-E and SSCI sections of the WoSCC database, which may restrict the data coverage. Although the WoSCC database is widely recognized for its rigorous assessment criteria and its extensive use in bibliometric research (74), important studies from other sources may still have been overlooked. Second, the inclusion of only English-language literature in this study may have resulted in the exclusion of high-quality, relevant studies in other languages. However, we supplemented our analysis with references cited in the publications.

## 5 Conclusion

In recent years, research on lactic acid in cancer has garnered significant attention and experienced rapid advancements. A bibliometric analysis of lactate and cancer-related publications over these nearly 10 years reveals the contributions and collaborations of countries, institutions, journals, and authors. Through an analysis of significant literature and keywords, we also propose potential changes and emerging trends in hot areas. Keywords such as ‘metabolic reprogramming’, ‘polarization’, ‘lung adenocarcinoma’, ‘tumor microenvironment’, ‘blockade’ and ‘chemodynamic therapy’ indicate that research is delving deeper, transitioning from traditional metabolic mechanisms to metabolic intervention, immune microenvironment regulation, combination of new therapeutic techniques and applications in specific cancer types. Research into lactic acid’s role in immune cell regulation, metabolism, and signaling pathways, combined with multi-modal imaging, big data analytics, and innovative drug delivery, is set to become a key trend in future studies, which promises new directions for identifying therapeutic targets, biomarkers, and developing advanced treatments.

## Data Availability

The original contributions presented in the study are included in the article/**Supplementary Material**. Further inquiries can be directed to the corresponding authors.
