# Supplementary material for: Research trends on lactate in cancer: a bibliometric analysis and comprehensive review (2015–2024)
Source: Front Immunol. 2025 May 9;16:1587867. doi: 10.3389/fimmu.2025.1587867 (PMC12098457; doi:10.3389/fimmu.2025.1587867)
Supplement: Supplementary file 1 [file Table1.docx]

**Retrieval strategy**

TS=(("Tumors" OR "Neoplasia" OR "Neoplasias" OR "Neoplasm" OR "Tumor" OR "Cancer" OR "Cancers" OR "Malignant Neoplasm" OR "Malignancy" OR "Malignancies") NOT ("tumor necrosis factor")) AND TS=(("Lactic Acid "OR "Lactate" OR "2-Hydroxypropanoic Acid" OR "2.Hydroxypropionic Acid" OR "Ammonium Lactate" OR "D-Lactic Acid" OR "L-Lactic Acid " OR " Sarcolactic Acid" OR "2Hydroxypropanoic Acid" OR "2Hydroxypropionic Acid") NOT ("Lactation" OR "Lactalbumin" OR "Lactobacillus" OR "Lactone" OR "Lactyl" OR "Lacrimal" OR "Lacuna" OR "Laccase" OR "Lacustrine" OR "Lacquer" OR "Lacquered" OR "Lactate dehydrogenase")) AND PY=(2015-2024) , with the article type limited to "Article" and the language restricted to English.
